# Flexible versus fixed spatial self-ordered response sequencing: Effects of inactivation and neurochemical modulation of ventrolateral prefrontal cortex

**DOI:** 10.1523/JNEUROSCI.0227-21.2021

**Published:** 2021-07-14

**Authors:** SFA Axelsson, NK Horst, Naotaka Horiguchi, AC Roberts, TW Robbins

**Affiliations:** 1 Department of Psychology, University of Cambridge, Cambridge CB2 3EB; 2 Behavioural and Clinical Neuroscience Institute, University of Cambridge, Cambridge CB2 3EB; 3 Department of Physiology, Development and Neuroscience, University of Cambridge, Cambridge CB2 3DY

## Abstract

Previously, studies using human neuroimaging and excitotoxic lesions in non-human primate have demonstrated an important role of ventrolateral prefrontal cortex (vlPFC) in higher order cognitive functions such as cognitive flexibility and the planning of behavioral sequences. In the present experiments, we tested effects on performance of temporary inactivation (using GABA receptor agonists) and dopamine D_2_ and 5-HT_2A_ receptor blockade of vlPFC via local intracerebral infusions in the marmoset. We trained common marmosets to perform spatial self-ordered sequencing tasks in which one cohort of animals performed 2 and 3 response sequences on a continuously varying spatial array of response options on a touch-sensitive screen. Inactivation of vlPFC produced a marked disruption of accuracy of sequencing which also exhibited significant error perseveration. There were somewhat contrasting effects of D_2_ and 5HT_2A_ receptor blockade, with the former producing error perseveration on incorrect trials, though not significantly impairing accuracy overall, and the latter significantly impairing accuracy but not error perseveration. A second cohort of marmosets were directly compared on performance of fixed versus variable spatial arrays. Inactivation of vlPFC again impaired self-ordered sequencing, but only with varying, and not fixed spatial arrays, the latter leading to the consistent use of fewer, preferred sequences. These findings add to evidence that vlPFC is implicated in goal-directed behavior that requires higher-order response heuristics that can be applied flexibly over different (variable), as compared to fixed stimulus exemplars. They also show that dopaminergic and serotonergic chemomodulation has distinctive effects on such performance.

## Introduction

Goal-directed behavior usually requires the planning of a self-ordered sequence of responses leading ultimately to reward, frequently in a spatial context. Although fixed response sequences may come under habitual control, self-ordered sequencing often demands response monitoring and strategies to reduce working memory load which engage the prefrontal cortex (PFC; Petrides and Milner, 1982; Owen et al., 1990). Hence, in the self-ordered spatial working memory task employed by Owen et al. (1990) humans searched on different trials through arrays of spatial locations associated with reward ‘tokens’ provided on a probabilistic basis and not replenished following choice. Those patients with lesions specifically to lateral (l)PFC regions exhibited performance decrements caused by repeated responding to previous locations, which were associated with an inefficient search strategy (Owen et al., 1990; Manes et al., 2002; Chase et al., 2008). A human functional imaging study (Owen et al., 1996) further showed that whereas dlPFC became active specifically during tasks with high spatial working memory load but minimal response sequencing requirement, ventrolateral (vl)PFC (i.e. area 47) exhibited activation specifically during self-ordered spatial response sequencing. This finding is consistent with findings from the disruptive effects of excitotoxic lesions of vlPFC (but not orbitofrontal PFC) of marmoset monkeys on a similar spatial search task in which they were required to self-order response sequences (of lengths up to 5) with variable stimulus locations on each trial (Collins et al., 1998; Walker et al., 2009). Unlike performance on a classical working memory task, spatial delayed response, PFC dopamine (DA) depletion failed to affect performance on the self-ordered sequencing task (Collins et al., 1998), and PFC serotonin (5-hydroxytryptamine, 5HT) depletion also failed to produce the perseverative deficits seen in marmosets with selective cell body lesions of the vlPFC (Walker et al., 2009).

**Figure 1:**
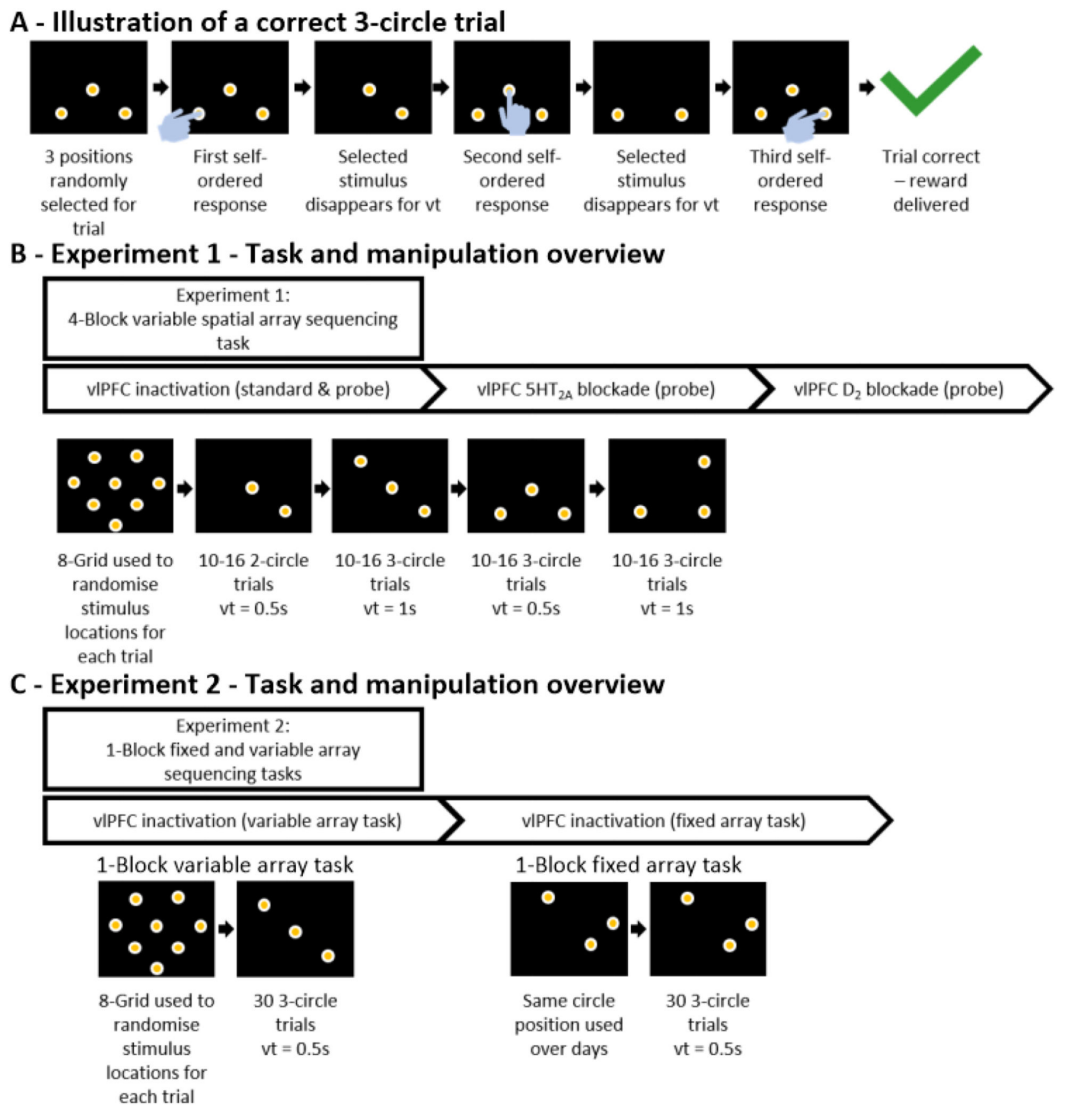
Experimental and task design. A) Visual representation of a correct 3-circle trial representative of all the self-ordered sequencing tasks. Once a location was selected it disappeared for the vt. Animals were allowed to continue responding during this time. B) Overview of vlPFC manipulations and task in experiment 1. For the 4-block task subjects performed a block of 2 circle trials (vt =0.5s) and 3 blocks of 3-circle trials with two different vt’s (1 & 0.5s). Circle positions were randomised based on an 8-stimulus grid. During probe sessions, as opposed to the standard task, errors were not punished by trial abortion. C) Overview of vlPFC manipulations and tasks in experiment 2. On the 1-block variable and fixed spatial array tasks animals only performed 3-circle trials with a vt of 0.5s. However, for the fixed array task, the same spatial configuration was presented on every trial and across every session.

While the self-ordered nature of response sequencing appears to be a critical requirement for engagement of the vlPFC, it is unclear whether the flexible manner in which this is required is also an essential task component. For example, self-ordered sequencing through a fixed, as opposed to variable, spatial array could become more habitual, hypothetically coming under alternative control by premotor cortex-striatal circuitry (Dezfouli and Balleine, 2012). Therefore, in this study we employed variable arrays of spatial locations for comparison with self-ordered performance of a fixed array. In addition, we sought to understand more clearly the nature of the performance decrements following vlPFC lesions in terms of the failure to disengage from repeating responses at the same location. To address these issues, the present study used a number of variants of the basic task (see Figure 1), following reversible inactivation of the vlPFC via infusions of GABA receptor agonists via permanently implanted cannulae. First, we employed a probe test on which errors did not immediately result in aborted trials. Second, in another cohort of animals we explicitly compared performance of self-ordered sequences in variable spatial arrays, presented both within and across sessions, versus fixed arrays presented identically both within and across sessions, which nevertheless allowed the marmosets to self-order their response sequences. Once animals had established consistent individual patterns of responding in the fixed array version, effects of inactivation were retested. In addition, using the probe task, we returned to the issue of possible monoaminergic modulation of sequencing performance by using acute microinfusions of the DA D_2/3_ receptor (-R) antagonist sulpiride and the selective 5HT_2A_-R antagonist M100907, in view of possible compensation following monoaminergic depletion in the earlier studies (Collins et al., 1998; Walker et al., 2009), to test the hypothesis that DA and 5HT may exert differential modulation over self-ordered sequencing.

## Materials and Methods

### Subjects

Eight common marmosets (*Callithrix Jacchus*), see Table 1, were bred on-site at the University of Cambridge marmoset breeding colony. The marmoset holding rooms were kept at a constant 24 °C with relative humidity of 55%. Holding rooms were gradually illuminated from 7.30 to 8.00 and gradually dimmed from 19.30 to 20.00, for a 12 h light/dark-cycle with 30 minutes of dusk/dawn. Cages (2.8x1.2x0.98m) contained a food tray, a nest box, wooden platforms at different heights and a variety of enrichment objects, including ladders, wooden branches and ropes. Five days a week, animals had access to water for 2 h after behavioral testing and during this time period were fed MP.E1 primate diet (Special Diet Services) and carrots. During weekends animals had *ad libitum* access to water and were fed a calorically equal diet consisting of bread, egg, rusk, fruit and nuts. All procedures were carried out in accordance with the UK Animals (Scientific Procedures) Act 1986 as amended in 2012, under project licences 70/7618 and P09631465. In addition, the University of Cambridge Animal Welfare and Ethical Review Body (AWERB) provided ethical approval of the project licence and its amendments, as well as individual studies and procedures via delegation of authorization to the NACWO for individual study plans.

### Apparatus

All behavioral testing was performed in a custom-built testing apparatus located in a separate room from the marmoset holding rooms. Animals were trained to enter a custom-made Perspex transport box (Biotronix, Cambridge, UK), in which they sat during testing. A door on the box was removed to provide access to a touch sensitive computer monitor (NEX121 TFT LCD Monitor, Nexio, Incheon, Korea). Animals had to reach through an array of vertical bar to respond to visual stimuli on the touch screen. Reward, in the form of banana milk (Nesquik banana powder in milk, Nestlé, York, UK), was delivered through a peristaltic pump to a licking spout accessible through the vertical bars. Auditory stimuli were presented through a speaker, out of sight of the subjects. Reward delivery and presentation of visual and auditory stimuli were controlled by the application MonkeyCantab (R. N. Cardinal), using the Whisker control system (Cardinal and Aitken, 2010).

### Behavioral Training

#### Pre-operative training

Subjects were trained to enter the transport box and habituated to the testing apparatus.

After successful habituation animals were familiarised with the liquid reward, learned the association between an auditory stimulus and access to reward and then acquired a touchscreen response for that reward; all previously described in Roberts et. al (1988). Subsequently, animals were trained on a spatial self-ordered sequencing task, in which they were required to select each of an array of identical stimuli presented on the screen, once only (see Figure 1A). Subjects were first trained to touch a stimulus, presented in a random spatial location for each trial, based on an 8-location grid. Once animals performed 20 trials in a session, task difficulty was gradually increased. The first step of subsequent training was the addition of a second identical stimulus in a distinct spatial location and animals were required to respond to both spatial locations, sequentially, in any order, in order to receive reward. Once a response was made to a stimulus, that stimulus disappeared for a set amount of time, denoted ‘vanishing time’ (vt). Animals were allowed to continue responding throughout the vt, but if they responded to the same stimulus more than once, the trial ended prematurely, the houselight was turned off for 5 s and the trial scored as incorrect. Vt was gradually decreased during training and the number of stimuli were increased until animals could perform twenty, 2-stimuli, followed by twenty, 3-stimuli trials (both with a vt=0.5s) with an accuracy of 80, and 50% respectively.

#### Post-operative testing

Three spatial self-ordered sequencing tasks were used for these experiments, see Figure 1. All of the tasks required subjects to perform a sequence of responses to 2 or 3 (depending on task) identical but spatially separated stimuli presented on a screen. For experiment 1, the 4-block task was designed to be similar to previous experiments, containing both 2 and 3 circle trials. This task was also used to investigate the chemical neuromodulation of performance. For experiment 2, the 1-block tasks were designed to allow us to contrast the effects of vlPFC manipulations on self-ordered sequences for variable versus fixed spatial arrays.

#### Experiment 1: 4-Block variable array spatial self-ordered sequencing task

Marmosets were tested on two versions of the 4-block variable sequencing task, the standard version and a probe version, which is a trial session designed to elicit incorrect responses. All four blocks consisted of the same number of trials, but the number varied between animals, from 10 to 16, as shown in Table 1, dependent on the total number of trials animals would perform consistently across sessions. In the standard version of the task, animals were required to successfully respond to each spatial location without repeating an already made response. Re-selecting an already made spatial response counted as an error and caused trial termination. Accuracy was therefore measured as the percentage of correct trials (or errorless sequences). Two types of errors are possible in this version of the task. An error can either be performed by repeating the immediately preceding response by responding to positions 1-1 or 1-2-2, termed a continuous perseverative error (after Sandson and Albert (1984)). An error can also be made by repeating the first response in the sequence instead of terminating the sequence by responding to positions 1-2-1, referred to as a recurrent perseverative error (Sandson and Albert 1984). In a probe session, animals were not punished for errors, but allowed to continue responding until they had selected each individual stimulus, at which point they received reward. All animals had experience with probe sessions on at least two separate sessions before the start of manipulations. A correct trial was still counted as an errorless sequence. However, in this probe version, unlike the standard version, repeated errors can occur on an incorrect trial and this allows further analysis of error type to be made. For example, an animal could make 20 errors in a session of 20 trials that were distributed over either (i) 10 incorrect trials or (ii) all occurring in one incorrect trial, perhaps reflecting a failure to respond to negative feedback. So, two additional measures of errors are presented. A total errors measure which is averaged across total trials (e.g., errors/trial) and a total errors measure averaged across incorrect trials (errors/incorrect trial) only. In the examples given above, errors/trial would be 1 for both (i) and (ii), whereas errors /incorrect trial would be 2 for (i) and 20 for (ii). Omissions occurred when the animals did not respond.

#### Experiment 2:1-Block variable array spatial self-ordered sequencing task

Three subjects performed a simplified version of the 4-block variable spatial self-ordered sequencing task; consisting only of 1 block with 30, 3-circle trials with a vanishing time of 0.5s. Errors through repetition of a response were punished, as before, by trial termination.

#### Experiment 2: 1-Block fixed array spatial self-ordered sequencing task

After completing manipulations on the 1-block variable sequencing task, parameters were changed to a fixed sequence version. Here, animals still performed responses to three stimuli in a self-ordered manner, with a vanishing time of 0.5s, as in the variable condition but the three stimuli were always in the same locations on every trial. After extended training (at least 10 sessions) on the same spatial trio, marmosets tended to perform a restricted set of alternative response sequences out of the total six distinct sequences that were possible. The spatial location of the three stimuli varied across testing, having been chosen based on each subject’s response sequences from the final two months performing the variable spatial self-ordered sequencing task. To ensure the fixed sequences were not already performed in a rigid fashion, and to allow responding to improve without reaching an immediate ceiling effect, the precise identity of the fixed sequence for each animal was based on three criteria: First, the subject had to have made at least 5 of the 6 possible correct response sequences during these two months. Second, the percentage of trials for which each correct response was performed was approximately equal, thus excluding prior response bias. Third, animals had to have an accuracy score for the specific fixed sequence of around 50%, which was considerably superior to chance performance (21%), but able to distinguish between impairments or improvements following manipulations.

### Surgery

Animals had permanent indwelling cannulae implanted to allow infusion of drugs into the vlPFC. For surgery, animals were pre-medicated with 0.1 ml of 100 mg/ml ketamine (Ketavet, Henry Schein, USA) and given prophylactic analgesic (0.03 ml of 50 mg/ml carprofen administered s.c.; Caprieve, Pfizer, UK) before being intubated and anaesthesia maintained using a mixture of vaporised isoflurane (Novartis animal health, UK) and O2 (2.25% Isoflurane in 0.3L/min O2). Animals were then placed in a marmoset stereotaxic frame (David Kopf, CA, USA). Anaesthesia was closely monitored clinically and by pulse oximetry and capnography.

Cortical depth was measured to allow for corrections of cannula targets, as previously described in Dias et al. (1997). A second depth check was performed bilaterally at anterior-posterior (AP) +17.25 to ensure depth was within range of 3.0-4.5 mm at an angle of between 8-10° (8°, n=1, 9°, n=5, 10°, n= 2). Double guide cannulae (Plastics One, Inc., Roanoke, VA, USA) were then inserted, at the same angle described for vlPFC depth check, with the caudal guide at AP +16.75 and the rostral guide at +17.75 at latero-medial (LM) ± 5.8. A surface reading was taken for the caudal guide and the cannula was lowered until it reached 1.2mm above the base of the skull, calculated from the vlPFC depth check. Guides were fixed in place by skull screws and dental acrylic (Simplex Rapid, Kemdent Works, Swindon, UK). Post-surgically, subjects were administered 0.18 ml of 3.8 mg/ml dexamethasone (0.09 ml injected into each quadricep) (Aspen Pharma Trading Ltd., Ireland). Subjects were also given analgesic once daily in the morning, for three days after surgery (meloxicam, 0.1 ml of a 1.5 mg/ml oral suspension; Boehringer Ingelheim, Ingelheim/Rhein, Germany). After surgery animals had *ad libitum* access to water for at least one week and were provided the food that was otherwise only available to them on weekends. Animals (n=3) performing the 1-block sequencing tasks were also implanted, in the same surgery, with cannulae targeting the caudate.

### Drug preparation and treatment

#### Drug treatment

For drug infusions, animals were gently restrained by a person other than the researcher and taken to a designated infusion-room. The researcher gently removed caps and dummies from cannula guides and cleaned the guides with injection wipes. For all infusions, an injector (Plastics One, Inc., Roanoke, VA, USA) was used that protruded +0.5mm from the cannula to allow for infusion at 0.7mm from the base of the brain. The injector was connected to a 10μl Hamilton syringe (701RN; Hamilton, Bonaduz, Switzerland) via PTFE tubing (0.3 mm diameter). Solvent flexible tubing was used to connect PFTE tubing to injector and syringe (0.38mm inner diameter, Elkay Laboratory Products, Ltd., Basingstoke, UK). Drug was accurately delivered by an infusion pump (KDS230, KD Scientific, Inc., Holliston, Massachusetts, USA). Injectors, tubing and syringes were all sterilised prior to setup.

#### vlPFC inactivation by infusion of GABA_A_ and GABA_B_ agonists

All animals in this study had a combination of muscimol (GABA_A_-receptor agonist) and baclofen (GABA_B_-receptor agonist) solution, referred to as ‘musbac’, infused into the vlPFC to allow temporary inactivation of the area. The drug solution was made up in saline to a concentration of 0.1 mM muscimol and 1mM baclofen before being filtered and aliquoted. Aliquots were stored at −20° C for a maximum of 3 months. Musbac was thawed immediately before infusion. Fresh sterile saline was used for the control vehicle infusion. The infusion was at a rate of 0.5μl per minute for 1 minute. A 25-minute pre-treatment time was allowed after infusion before testing.

#### Intra-vlPFC D_2_ receptor blockade using sulpiride

(S)-(-)-Sulpiride (sulpiride) (Sigma Aldrich, UK) is a relative selective D_2_/3 dopamine receptor antagonist (Kohli and Cripe, 1979; Pauwels et al., 1993; Ago et al., 2005). It was prepared in two different concentrations, 3.75 μg/μl and 2.5 μg/μl. The drug was dissolved in 4000 μl of 0.1M HCl in saline. Solution was neutralised by slow addition of 1M NaOH until pH reached 7. Stock solution was diluted with phosphate-buffered saline (pbs) until a concentration of 10 μg/μl was achieved (a target volume of 10000 μl). Stock solution was filtered, aliquoted and stored at −20° C for a maximum of 2 weeks. Stock solution aliquot was thawed, diluted with pbs and filtered to desired concentration (3.75 or 2.5 μg/μl) before infusion. Vehicle was treated in an identical fashion but sans drug.

Sulpiride (and corresponding vehicle) was infused at a rate of 0.5 μl/min over 1 minute. A 10 minute pre-treatment period was allowed before animals were tested. All animals treated with sulpiride were treated with both doses of drug and the vehicle.

#### Intra-vlPFC 5-HT_2A_ receptor blockade using M100907

MDL-100,907 (M100907) (Sigma Aldrich, UK) is a selective 5-HT_2A_ receptor antagonist (Kehne et al., 1996). It was prepared in four different concentrations (0.5, 1, 1.5 and 2 μg/μl). M100907 was made fresh before each infusion. The desired amount of drug was dissolved in 40 μl 0.1M HCl and dissolved to a volume of around 1000μl using pbs. Vehicle was 40μl 0.1ml HCl dissolved in 960 μl pbs. M100907 (and corresponding vehicle) was infused at a rate of 0.5 μl/min for 1 or 2 minutes, depending on dose. A pre-treatment time of 12 minutes was allowed after infusion, prior to testing. A range of doses, see Table 2, of M100907 was used between animals until they reached the maximum dose (2 μg) or a dose which caused them to disengage from testing. Disengaging was classified as having performed fewer than 50% of trials in block 4. All animals had 0.5 μl infusions prior to 1 μl infusions. The reason for the increase in volume rather than concentration was because of drug solubility.

### Experimental design, measurements and statistical analysis

The general design was that animals performed the test Monday – Friday every week, at approximately the same time each day. All experiments used a within-subject study design; for an overview see Figure 1. An infusion was performed towards the end of the testing week, if animals had shown stable responding during the week. In general, only one experimental drug infusion was performed per week and each drug infusion was tested on one session only.

In Experiment 1 (number of subjects =5), the first infusion was musbac or vehicle on the probe task. Inactivation using musbac was also investigated on the standard task (number of subjects =4). Following inactivation on the probe, marmosets (number of subjects =4) received infusions of either D_2_ receptor antagonist sulpiride or 5-HT_2A_ receptor antagonist M100907 on the 4-block probe task; with the order of the two counterbalanced between subjects.

In Experiment 2 (number of subjects=3) after identical pre-operative training, marmosets were tested on a simplified version of the 4-block task, consisting of only 1 block, with 30, 3-circle trials with a vt of 0.5s. Following infusions of musbac and vehicle on this task, marmosets acquired and performed the fixed sequence task before receiving infusions of musbac and vehicle into the vlPFC again.

Measurements included trials completed, accuracy, errors per trial and errors per incorrect trial. Trials completed indicated the percentage of trials in which animals performed a correct or incorrect sequence and did not refrain from responding for 60 s. Accuracy was the number of sequences performed without errors. Errors per trial was the number of errors performed divided by the number of correct plus incorrect trials, while errors per incorrect trial were the number of errors divided by incorrect trials.

Testing data were collected in a Microsoft Access database. Data were exported into Microsoft Excel (Office 365) and R studio (Version 1.2.1335, RStudio: Integrated Development for R. RStudio, Inc., Boston, MA). Different statistical tests were performed but P < 0.05 was used for statistical significance for all tests.

#### Inactivation of vlPFC on the spatial self-ordered sequencing task

Statistical analysis and graphical representation were performed in GraphPad Prism (Version 7.03 for Windows, GraphPad Software, La Jolla, California, USA). Data were presented as mean values with the standard error of the mean (SEM). In Experiment 1, a two-way repeated measures analysis of variance (ANOVA) was performed on the 4-block task with post-hoc tests using Sidak’s correction for multiple comparison. In Experiment 2, a two-tailed paired t-test was performed on data from the variable and fixed sequencing tasks independently. They were also analysed using a two-way ANOVA with an additional factor of fixed-variable task.

#### D_2_ and 5HT_2A_-receptor blockade on the 4-block spatial self-ordered sequencing task

Statistical analysis was performed in RStudio. Parts of the dataset were transferred from RStudio to create graphs in GraphPad Prism (Version 7.03 for Windows, GraphPad Software, La Jolla, California, USA). Data were analysed using multiple linear mixed effects models with the R package ‘lme4’ (Bates et al., 2015; Boisgontier and Cheval, 2016). Dose and block were fixed effects and subject was a random effect. ANOVA was performed on the model to acquire p-values. All doses for each animal were included in the analysis. For M100907 both replicate doses of 1 μg and both vehicle infusions, were included in the model. Data were presented graphically for each subject individually alongside the average across all animals. To enable easier reading of graphical representations of the M100907 data, the doses that were replicated (1 μg of M100907 and the two vehicle infusions) were presented as mean values.

### Histology

Histological analysis was used to assess cannulae placement. Animals were pre-medicated with ketamine and placed into an incubator for five minutes before being injected with 1ml of 200mg/ml solution of pentobarbital IV (Dolethal; Merial Animal Health, Essex, UK). Loss of heartrate was confirmed using a stethoscope before animals were perfused transcardially with 300ml 0.1 M phosphate buffered saline, followed by 300 ml 10 % solution of formalin stabilised in phosphate buffer. The brain was removed and placed into 10% formalin solution for 24 hours before being transferred into a 30% W/V sucrose solution for at least 48 hours. Brains were sectioned using a microtome (40 μm coronal sections) before being mounted on slides and stained using Cresyl-violet. Slides were viewed under a Leitz DMRD microscope (Leica Microsystems) and cannula placements were drawn onto a schematic containing a series of standard outlines of the marmoset brain through the prefrontal cortex.

The volume used to infuse the drugs was relatively small in relation to the large area of the vlPFC and we provide histological data to indicate that the cannulae tips for each monkey were accurately placed in the central regions of the lPFC. Using staining and histological methods it is not possible to determine the spread of an infusion. However, the consensus from previous studies using fluorescent-tagged (Allen et al., 2008) or radio-labelled (Sperber et al., 1989; Krupa and Thompson, 1997; Martin and Ghez, 1999) muscimol and/or glucose metabolism (Martin and Ghez, 1999) is that there is an effective radius of ~1.5mm for the inactivating effects of muscimol at doses, volumes, and infusion rates similar to those used here.

## Results

### Histological analysis

**Figure 2:**
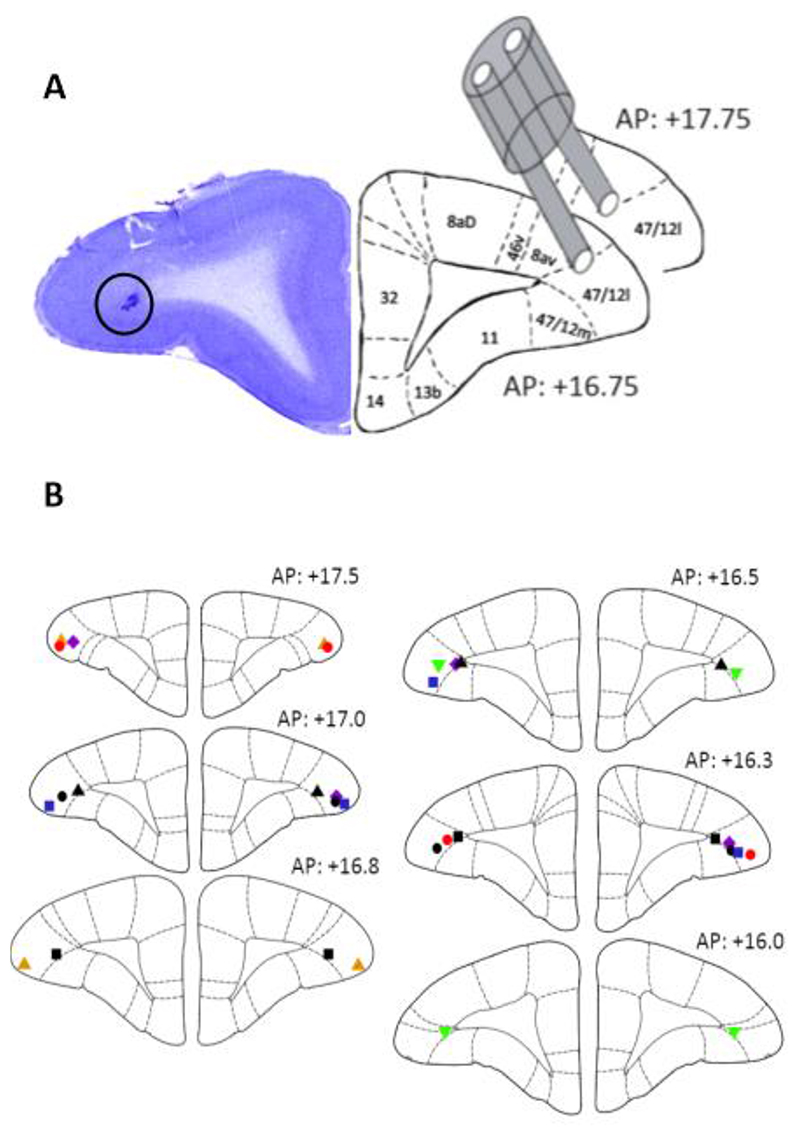
Cannula placements for experiment 1 and 2. A) Left side, example photomicrograph of a Cresyl-violet stained section from this experiment, infusion site marked by a circle. Right, Schematic of vlPFC cannula target area. Given the rostro-caudal extent of vlPFC, double cannula, 1mm apart anteroposteriorly, were used in each hemisphere. Injectors protruding 0.5mm from the cannulae were used, allowing for infusions at 0.7mm from base of brain in area 47/12. B) Actual cannula placements for individual subjects marked with respective symbols.

The cannulae were confirmed to have targeted the vlPFC in all marmosets, as seen in an example photomicrograph of a Cresyl-violet stained section at the level of the prefrontal cortex (Figure 2A) as well as summary schematics (Figure 2B). In most animals, the infusion location encompassed both area 47/12l and 47/12m.

### Experiment 1:4-Block variable array sequencing tasks

#### Effects of temporary inactivation of vlPFC

##### Standard test

**Figure 3:**
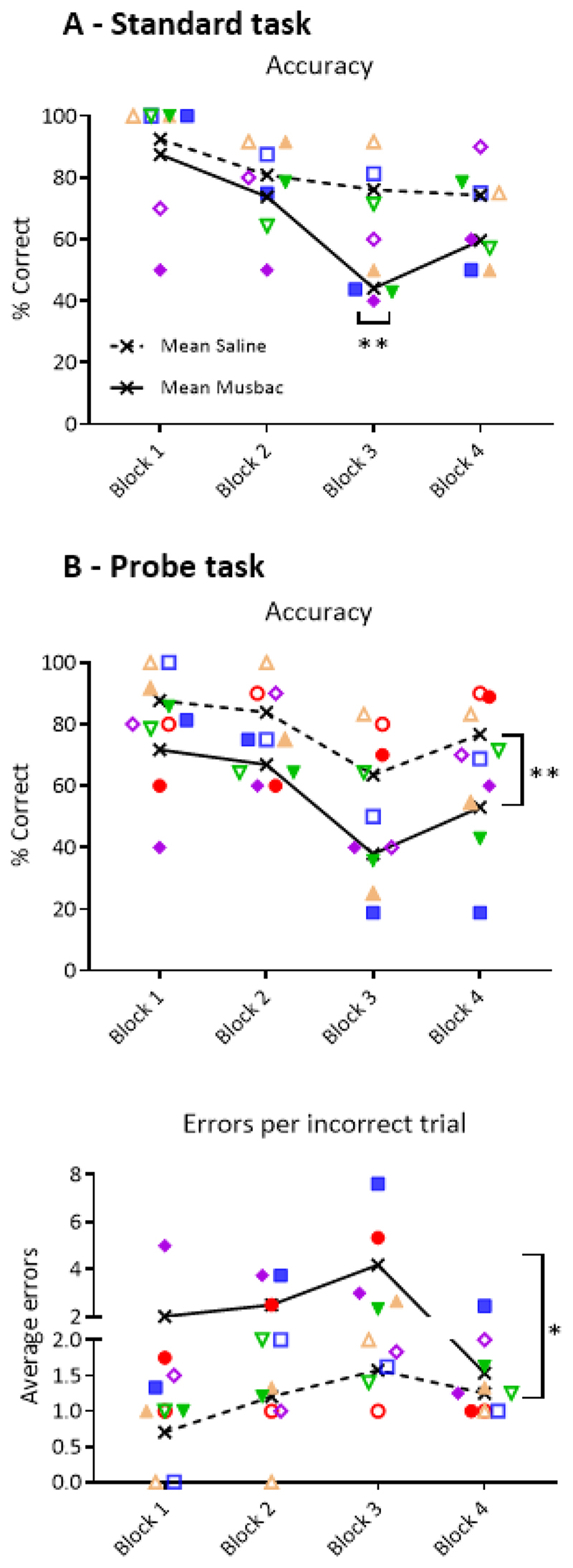
Effect of inactivation of vlPFC on the standard and probe 4-block variable sequencing task. Graph shows the mean accuracy per block for subjects after infusion of saline (dashed line) and musbac (solid line) into the vlPFC. Individual data points are presented as a unique coloured symbol, where musbac data points are filled, while saline datapoints are hollow. A) For the standard task, inactivation of vlPFC impaired performance only in the third and most difficult block of the task. B) On the probe-task, inactivation decreased accuracy across all blocks. Inactivation also increased the number of errors performed on incorrect trials in the probe task.

Inactivation of the vlPFC using musbac impaired task performance in the critical third and most difficult block of the task (Figure 3A). ANOVA revealed a significant interaction between block and musbac treatment (F(3,9)=3.895, p= 0.049) with post-hoc tests revealing a significant effect in block 3 (p=0.006). Additional analysis was performed to understand if treatment with drug affected the distinct error types differentially. A three-way repeated measures ANOVA revealed that there was no interaction between drug and error types on performance (F(1,2)=3.728, p= 0.193), nor was there an interaction between drug, block and error type (F(2,4)=1.439, p=0.338). Musbac did not affect total trials completed (F(1,3)= 0.009, p= 0.931).

Five different latencies were also investigated:

- Trial time; from stimulus presentation to completed sequence or trial errors
- Initiation time; from stimulus presentation to first response
- Performance time; from first to last stimulus selection
- Inter-response times; from first to second and second to third stimulus selection

The performance time across all blocks were faster under control conditions, average median performance time, 646.5, 1391, 1329 and 1573 ms for block 1 to 4 respectively, with a mean difference compared to inactivation of 129.4, 73.25, 90.24 and 147.3 with a standard error (SE) difference of 116.7. Analysing the increased median performance time using a two-way repeated measures ANOVA revealed that effect was only trending (F(1,3)=9.336, p=0.055). The increased performance time was likely driven by a significant increase in the median inter-response time for the first to second response (F(1,3)=10.21, p=0.049). Average median time for first to second response, 641.9, 598.3, 613.3 and 727 ms for block 1 to 4 respectively, with a mean difference compared to inactivation of 129.9, 55.4, 22.6, 150.1 with a SE difference of 52.3. No other latencies showed significant differences (p > 0.05).

##### Probe test

Inactivation of vlPFC with musbac impaired sequencing in all blocks of the probe task, in which errors were not punished (Figure 3B). Musbac infusions significantly reduced accuracy and increased the number of errors per trial as well as the number of errors per incorrect trials. A two-way repeated measures ANOVA showed that there was a main effect of musbac on accuracy (F(1,4)=38.51, p=0.003), on the average number of errors per trial (F(1,4)=23.64, p=0.008) and the number of errors per incorrect trial (F(1,4)=10.61, p=0.031). Further analysis was performed to understand if inactivation affected error types differentially. A three way repeated measures ANOVA revealed that there was an interaction between drug and error type (F(1,4) 12.399, p=0.024). This analysis was followed up by investigating individual error types in separate two-way ANOVAs. Analysis revealed that there was a main effect of musbac on recurrent perseverative (1-2-1 response) errors (F(1,4)=8.49, p=0.044), but not on continuous perseverative (1-1 or 1-2-2 response) errors (F(1,4)=5.568, p=0.076). Thus, when the vlPFC was inactivated, marmosets were less accurate, increased the number of errors they made per incorrect trial, and committed relatively more recurrent perseverative errors, as compared to vehicle. However, musbac did not affect the overall engagement of marmosets on the task, with only subject 3 making a single omission in the fourth block when treated with musbac.

#### Effects of intra-vlPFC 5HT_2A_ receptor blockade

**Figure 4:**
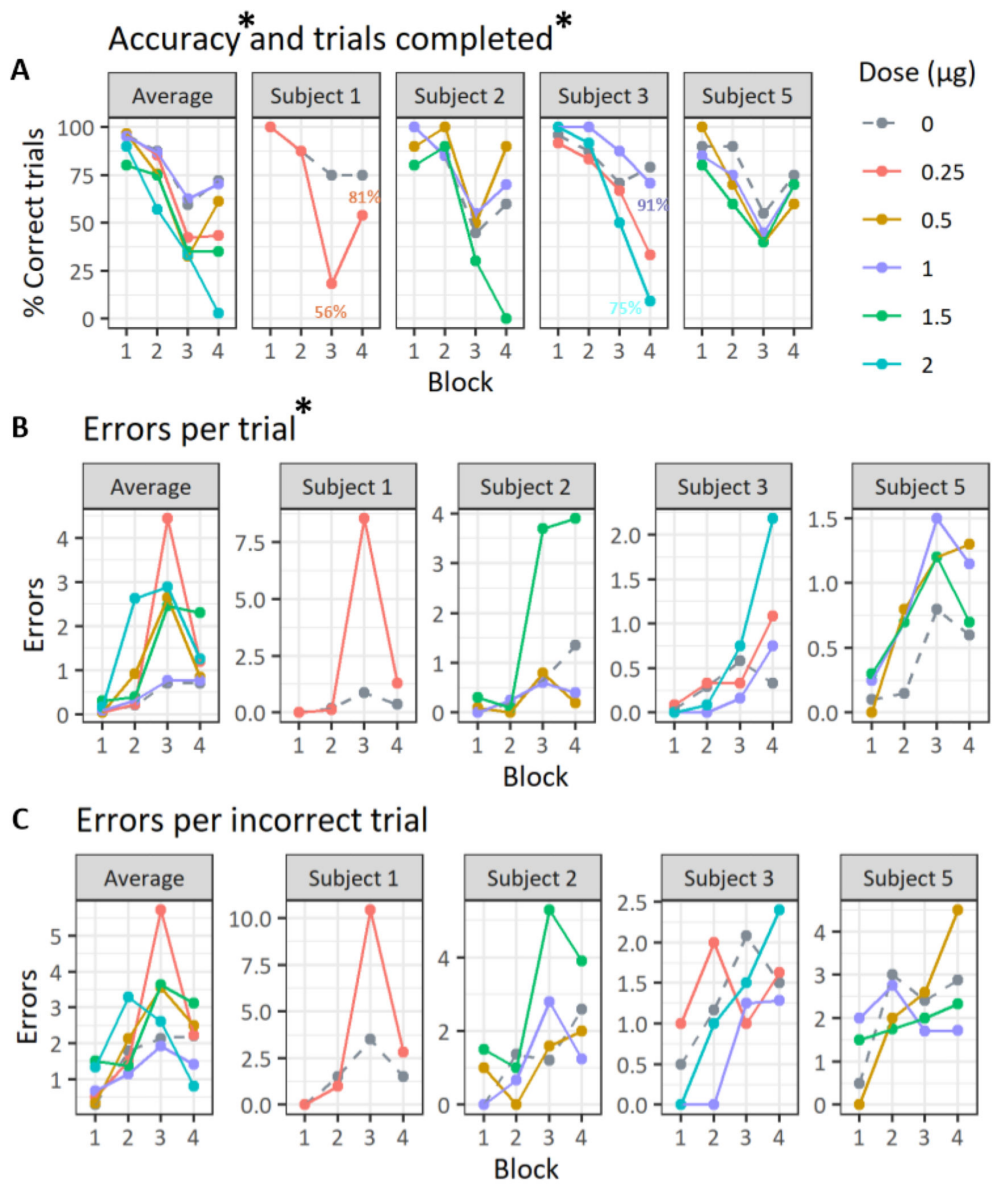
Behavioral performance of the self-ordered spatial sequencing task following vlPFC 5HT_2A_-R blockade. The leftmost graph show mean performance across all subjects, but note that points are averaged on only two values in some cases. All graphs have the same color coding for dose. * in title denotes a main effect of treatment. Replicate doses of vehicle (dose 0) and dose 1 are presented as a mean. A) Points show the percentage of completed trials where a sequence of three was performed without any error. If an animal omitted (by not making a response for 60 s) the trial was counted as not completed. If any trials were omitted in a block, the percentage of trials completed for that block is presented next to the accuracy value B,C) Points with corresponding lines show the average number of errors made on all trials, completed with and without errors (B), or only on trials performed with errors (C).

Blockade of 5HT_2A_ receptors by infusion of M100907 into vlPFC on the Probe test impaired performance of self-ordered spatial response sequences see Figure 4, as reflected by a significant reduction in accuracy and increased numbers of errors per trial. The number of errors per incorrect trials were however not significantly different. The highest doses of M100907 for subjects 1, 2 and 5 were excluded from the analysis due to animals disengaging from testing altogether. At their individually highest dose, subjects 1, 2 and 5 all performed ≤ 30% of the trials in the fourth block. Analysis of accuracy for other doses using a linear mixed effect model revealed a main effect of drug on accuracy (F(5,52.572) = 3.583, p= 0.007), where M100907 decreased accuracy. Qualitatively, this effect was strongest in the third and fourth blocks of the task, where accuracy was drastically decreased. On trials completed, there was a significant effect of drug (F(5,53.235) = 3.793, p = 0.005) and block (F(3,51.403) = 5.728, p = 0.001) as well as an interaction between the two (F(15,51.403) = 3.491, p < 0.001). Only two animals made omissions at the doses included in the analysis. On errors per trial there was also a significant effect of drug (F(5,49.083) = 2.4487, p = 0.046) and a significant effect of block (F(3,53.103) = 8.6147, p < 0.001). In 3 out of 4 subjects there was an increase in errors per trial in the third block of the task. On the fourth block this effect was true for all animals. On number of errors per incorrect trials, there was no significant effect of drug (F(5,53.769) = 1.289, p = 0.282). Investigating individual error types (i.e., continuous versus recurrent perseveration) by addition of error type as a fixed effect in the model revealed no interaction between drug and error type (F(5,108.5)=0.501, p=0.775).

#### Effects of intra-vlPFC D_2_ receptor blockade

**Figure 5:**
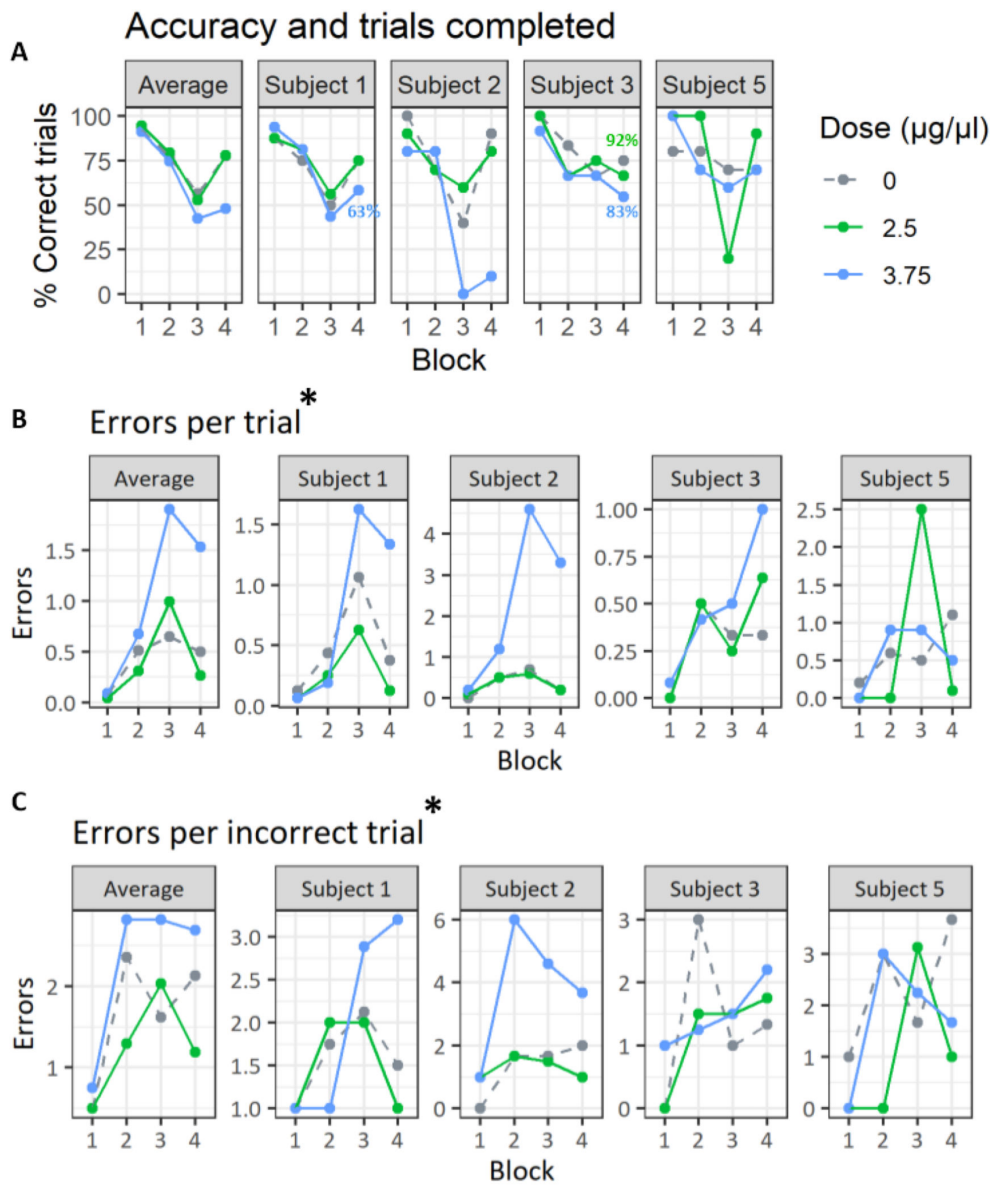
Behavioral performance of the self-ordered spatial sequencing task following vlPFC D_2_-R blockade. The leftmost graph show average across all subjects. All graphs have the same color coding for dose. * in title denotes a main effect of treatment. A) Points show the percentage of completed trials where a sequence of three was performed without any error. If an animal omitted (by not making a response for 60 s) the trial was counted as not completed. If any trials were omitted in a block, the percentage of trials completed for that block is presented next to the accuracy value B,C) Points with corresponding lines show the average number of errors made on all trials, completed with and without errors (B), or only on trials performed with errors (C).

Blockade of intra-vlPFC D_2_-R by infusion of sulpiride impaired performance of the spatial response sequencing task, see Figure 5, as reflected by the significantly increased numbers of errors per trial and errors per incorrect trials. The linear mixed-effects models showed that there was a main effect of treatment on errors per trial (F(2,33)=4.089, p= 0.025) and block (F(3,33)= 5.036, p= 0.006), with no interaction between the two (F(6,33)= 0.096, p= 0.462). All animals showed an effect of increased errors per trial in the third and/or fourth block. Sulpiride also significantly increased the numbers of errors per incorrect trials, (F(2,33)= 4.192, p= 0.023). The effect of drug on errors per incorrect trials was consistent across animals in the third block of the task and in three out of four animals in the fourth block. Accuracy was numerically affected for some animals, in some blocks, but not significantly impaired. The linear mixed effects model did not show a significant effect of treatment with sulpiride on accuracy (F(2,33)= 3.074, p=0.059) and there was no interaction of block and treatment (F(6,33)= 0.914, p= 0.496). An accuracy decrease was very clear in two subjects (subject 5 and 2), but effects in the other two animals were less evident. Investigating individual error types (i.e., continuous versus recurrent perseveration) by addition of error type as a fixed effect in the model revealed no interaction between drug and error type (F(2,69)= 0.144, p=0.985).

### Experiment 2: Contrasting variable and fixed array sequences

Direct comparison of inactivation on the variable and fixed array sequence versions using a two-way repeated measures ANOVA revealed an interaction between treatment and task (F(1,2)=21.06, p = 0.04), indicating that there was a difference in the effects of inactivation on the two tasks, each task was thus analysed separately.

**Figure 6:**
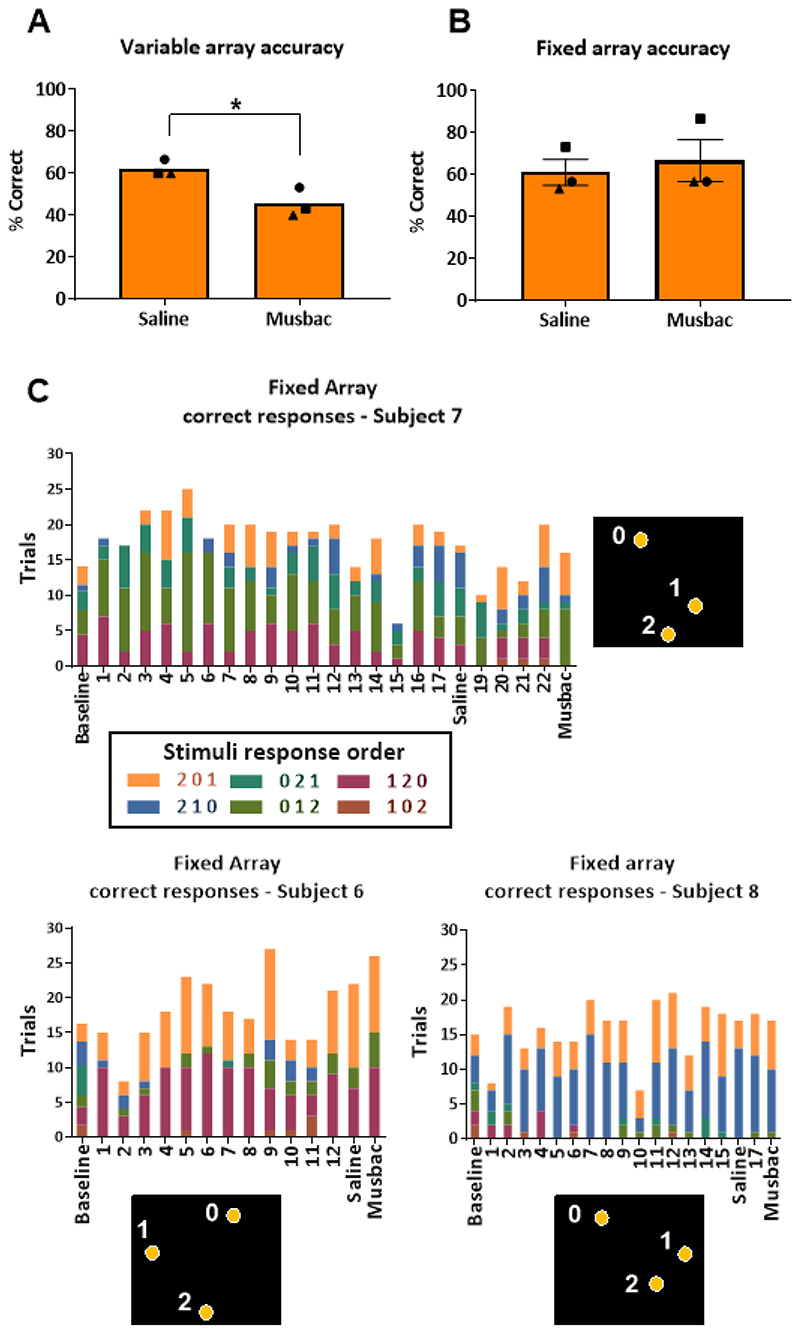
Performance of the variable and fixed array spatial self-ordered sequencing tasks. A,B) Graph shows the mean with SEM accuracy for groups on either the 1-block variable array task (A) or the 1-block fixed array task (B). A) Inactivation of vlPFC impaired performance of the sequencing task with variable arrays, replicating findings from the 4-block task. B) Once the subject was performing a constrained set of responses to solve the sequence, vlPFC inactivation no longer impaired performance. C) Correct responses on the 1-block self-ordered sequencing task with a fixed array across all sessions. Each different colored bar segment represents the number of trials that a particular correct sequence was performed in a given session for subject 6, 7 and 8. The height of the full bar provides a measurement of total correct trials out of 30. The fixed array for the subject, with the number corresponding to each spatial location, and the potential response sequences with respective color codes are presented next to the graphs. The baseline data point represents the distribution of responses when the particular spatial array was presented at random on the variable sequencing task for two months prior to starting the fixed array task, converted into a 30-trial representation. It can be seen that following extended practice with a particular spatial array, a reduced number of sequences were employed as reflected by the reduced number of colours in the histogram plots across sessions.

The 1-block variable array sequencing task was designed to consist only of the trials showing a significant impairment in the standard version of the 4-block task, presented above. Replicating the finding above, the new cohort of marmosets in Experiment 2 (n=3) showed impaired performance of this modified task following musbac-induced inactivation of vlPFC (Figure 6A). A two-tailed paired Student’s t-test showed that there was a significant effect of musbac on accuracy, mean ± SEM difference for Musbac − Saline = −16.67 ± 1.923, p=0.013.

As expected, based on the criterion for individual selection of the fixed array sequence of three stimuli, all three marmosets showed a wide range of response sequences when first solving the fixed array sequence task. In all cases, however, the range of response sequences narrowed somewhat across training, so that by the end they were performing fewer sequence options, see Figure 6C. Further breakdown of the correct responses indicated that all three animals adopted a strategy to solve the sequence, but only two out of three consistently performed it over many days. One animal, subject 6, adopted a strategy by solving the sequence through moving across the screen in a counterclockwise fashion. Another animal, subject 8, adopted a strategy where she almost exclusively started by responding to a stimulus in a specific position, before responding to either of the other two stimuli. The final subject, 7, adopted a clockwise strategy for ten testing days before again showing greater flexibility in his responding.

In contrast, however, to the variable array sequencing task, musbac-induced inactivation of vlPFC no longer impaired task performance on this fixed array sequence version (Figure 6B). Inactivation was without effect on accuracy; mean ± SEM difference for Musbac-Saline = 5.56 ± 4.008, p=0.299 (two-tailed paired t-test).

### Summary of results

A causal role for vlPFC in performance of variable array spatial self-ordered sequences was demonstrated in two separate cohorts of marmosets performing the 4-block and 1-block task, respectively. We showed that transient local inactivation of the vlPFC using infusion of GABA agonists decreased the number of trials performed correctly while also increasing the number of errors made on incorrect trials. This effect was behaviorally specific in that there were no deficits produced by inactivation on fixed array self-ordered sequencing. These findings were followed up by investigations into the chemical neuromodulation of performance. Infusion of 5HT_2A_ or D_2_ receptor antagonists into the vlPFC both impaired performance by increasing errors, although blockade of the two receptors significantly impaired distinct measures. Blockade of 5HT_2A_ receptors increased the number of errors by decreasing accuracy, as compared to vehicle, but did not significantly increase the number of errors made on incorrect trials. Blockade of D_2_ receptors did not significantly affect accuracy, but significantly increased the number of errors by increasing the number of errors performed on incorrect trials.

## Discussion

These findings demonstrate that reversible inactivation of vlPFC induces a highly selective deficit in the performance of flexible, but not constrained, sequences of spatially self-ordered responses. An innovative control procedure tested marmosets’ self-ordered sequencing performance on a fixed spatial array, finding no deficits following vlPFC inactivation. Therefore, the impairment in self-ordered sequencing following vlPFC inactivation was confined to situations when flexible responding to a variable spatial array of search options was required. This is consistent with a likely strategic role of vlPFC to produce generalised abstract performance rules when responding to different spatial arrays or stimulus sets to achieve flexible, goal-directed behavior. Previous studies contained a short delay component in the sequencing task, increasing working memory load, but we demonstrated that the impairment is present even in the absence of a delay after each response, further emphasizing a likely role for vlPFC in sequencing rather than working memory (Owen et al., 1996), and in line with previous findings showing that vlPFC is not required for maintaining information online during a delay (Rushworth et al., 1997). However, as hypothesised previously (Walker et al., 2009) for effective sequencing performance it is necessary to resist distraction and vlPFC lesions may disrupt performance by impairing attentional control. Further experiments defined how vlPFC controlled performance using task variants that enabled detailed error analysis in terms of the distribution of errors over trials and the continuous or recurrent nature of the perseverative behavior. On the probe test version, while vlPFC inactivation both impaired sequence accuracy and enhanced recurrent perseveration in terms of repeated errors on the same trial following an error, effects of relatively selective neurochemical modulation via D_2_-R (sulpiride) or 5HT_2A_-R (M100907) blockade had distinct performance profiles, with impairments in sequence accuracy only following M100907 infusions and increased perseveration on incorrect trials after sulpiride.

### Effects of vlPFC inactivation on self-ordered sequencing

The largest impairment following inactivation was on the probe-version of the 4-block task, which had the highest requirement for flexibility in responding. Accuracy was decreased and the frequency of errors performed on incorrect trials was also higher than following control saline infusions. This indicates that not only is vlPFC responsible for planning successful self-ordered response sequences, but also that once the plan is lost following inactivation, animals were unable to adapt their performance to rescue excessive superfluous responding. However, an impairment was also evident in the standard 4-block version of the task (errors terminating the trial) and subsequently replicated in a new cohort of marmosets in the 1-block version (see Figure 1). The latter result indicates that the deficit in 4-block task performance was not simply one of failing to adapt to the different sequence requirements, involving changes in the number of spatial locations and vanishing times. Following over-training with the fixed spatial array version all animals developed individual, heuristic strategies for solving this version that generally resulted in a narrowing of the response sequences employed (Figure 6). Inactivation was without effect on this fixed-array version. The selective effect therefore on variable response sequencing suggests that the vlPFC is not required for executing self-ordered response sequencing per se, implying a role for the vlPFC in flexibly guiding behavior in variable situations where a general rule, principle or heuristic needs to be derived and applied to a larger set of problems. This conclusion is compatible with the findings of Procyk and Goldman-Rakic (2006) on the role of the dorsolateral PFC in the self-organisation of behavior. More generally, it concurs with findings on reversal learning with different stimulus sets (Rygula et al., 2010), visuomotor learning tasks (Puig and Miller, 2012, 2015), and other forms of strategy implementation (Bussey et al., 2001; Baxter et al., 2009) that also suggest vlPFC to be primarily required for adapting performance heuristics or rules to new situations or stimulus sets.

This leaves open the question of which neural structures and circuitry might be implicated in initially selecting and then performing preferred sequence(s), as in the fixed condition, a likely candidate being the basal ganglia (e.g. Yin et al., 2009; Wymbs et al., 2012; Jin et al., 2014).

#### Effects of intra-vlPFC dopamine D_2_ and serotonin_2A_ receptor blockade

The significant neuromodulatory effects in the present study stand in contrast to earlier failures to find significant effects of PFC dopamine or serotonin depletion on response sequencing (Walker et al 2009), which may have been due to the well-known capacity of depleted monoaminergic systems to exhibit functional compensation (Björklund et al., 1973; Robinson et al., 1990; Collins et al., 2000; Man et al., 2010), avoided by the present acute manipulations. However, the present manipulations targeted specific receptors (D_2_ and 5-HT_2A_) and in both cases there is evidence of opponent functional interactions between D_1_ and D_2_ dopamine receptors on the one hand (Durstewitz and Seamans, 2008) and 5-HT_2A_ and 5-HT_2C_ receptors on the other (Winstanley et al., 2004). Thus, our acute manipulations likely disrupted the functional balance that normally exists in these pathways, an effect less likely to have occurred as a result of removing the dopamine and serotonin innervation.

The present deficits following infusions of the D_2_/3-R antagonist sulpiride are probably attributable to an effect on D_2_-R, because of low expression of D3-R in cortex (Levesque et al., 1992- though see Clarkson et al., 2017) whereas D_2_-R are primarily located on prefrontal layer V pyramidal cells (Lidow et al., 1998; Santana et al., 2009) rather than on presynaptic DA terminals (Vijayraghavan et al., 2016, 2017). The significant increase in errors though did not consistently, significantly affect task accuracy, per se. Instead, errors tended to accumulate on incorrect trials indicating that the effect was primarily in error correction. Findings following microiontophoretic application of D_2_ agents to the dlPFC in rhesus monkeys performing an oculomotor spatial working memory task showed that D_2_-R activity was related to motor saccades performed rather than being related to delay (Wang et al., 2004). A similar response-related activity has been detected in the vlPFC (Puig and Miller, 2015) and has been suggested to relate to processing of motor feedback (Wang et al., 2004; Arnsten et al., 2015). Our finding of repetitive errors on the same trial following intra-vlPFC sulpiride is hence consistent with a blockade of inhibition of pyramidal cell function via D_2_-R. The findings are compatible with the hypothesis that activity in D1 and D_2_-R may be functionally opposed in maintaining stability of PFC ensemble firing, D_2_-R antagonism therefore potentially promoting perseveration (Durstewitz et al., 2000).

By contrast, 5HT_2A_-R antagonism significantly affected response accuracy. This may be consistent with the location of 5HT_2A_-R on the apical dendrites of pyramidal cells, close to the soma (Jakab and Goldman-Rakic, 1998), especially in layers III and V of the PFC (de Almeida et al., 2008).

Mechanistically, these receptors have been suggested to amplify glutamatergic excitatory synaptic currents (Marek and Aghajanian, 1999), hence a 5HT_2A_-R antagonist could be expected to reduce such currents. Previous evidence of behavioral effects following 5HT_2A_ manipulation intra-l-PFC has been scarce, although a complex role in working memory has been suggested (Williams et al., 2002). We hypothesise that the present impairment following vlPFC infusion is a result of pyramidal 5HT_2A_ receptors being unable to amplify task relevant sensory inputs, and hence guide a behavioral plan. Error correcting, D_2_ receptor-related mechanisms might nevertheless still be intact, explaining why the errors per incorrect trials were not significantly increased. However, we emphasize that these drug treatments did not produce completely dissociable effects on performance and is, for example, possible that one of the sequelae of the blockade of D_2_ receptors on GABA interneurons is on the downstream regulation of 5-HT activity, as occurs following intra-striatal sulpiride (Nakazato et al., 1998).

#### Implications

The present findings are of relevance to mental health disorders such as schizophrenia and obsessive-compulsive disorder (OCD). For OCD patients, planning deficits involving flexible self-ordered spatial sequencing are correlated with decreased resting state and functional connectivity between the lPFC and putamen (Vaghi et al., 2017a,b). For schizophrenia, similar dysconnectivity between lPFC and basal ganglia has been shown (Zhou et al., 2007) as well as impairments in planning and sequencing tasks with heritable components apparently independent of IQ (Lemvigh et al., 2020).

Antipsychotics targeting D_2_ and 5HT_2A_-Rs are the first line treatment for schizophrenia. Recovery of cognitive functioning, including restored performance of action sequences, is important for long-term community outcome for patients (Green et al., 2000, 2004; Semkovska et al., 2004). Our findings that blockade of 5HT_2A_ or D_2_-R impaired sequencing may indicate that current treatments for schizophrenia could impede successful community outcomes. Indeed, it has been reported that patients treated chronically with antipsychotics having high affinity for the 5HT_2A_-R show decremental planning performance (Tyson et al., 2004). Moreover, systemic treatment with sulpiride impairs sequencing performance in healthy volunteers (Mehta et al., 1999). Drug-naïve OCD patients show reduced 5HT_2A_-R availability within the l-PFC (Perani et al., 2008), indicating that the reduction in these receptors might impair behavioral planning, supported by recent evidence that treatment with selective serotonin reuptake inhibitors specifically improves planning performance in OCD (Lochner et al., 2020).

#### Methodological considerations and limitations

This study used an established method for inactivating cortical areas with a mixture of GABA-receptor agonists. The possibility of diffusion from the site of infusion appears slight in relation to the overall volume of the target region and the behavioral effects were consonant with effects of focal vlPFC excitotoxic lesions (Walker et al., 2009).

The order of the variable and fixed sequencing tasks was not counterbalanced because of likely transfer difficulties for marmosets trained initially on fixed array sequences and then shifted to variable sequences (given the extensive training required to stabilise performance with no prior fixed array sequence training). Although it could be argued that the lack of impairment following vlPFC inactivation in the fixed array sequencing task was due to drug tolerance or learned compensation, significant effects of inactivation were evident for infusions on both test occasions in Experiment 1, standard and probe versions, arguing against these possibilities.

#### Summary and Conclusions

Inactivation of the vlPFC in marmosets produced a selective deficit on self-ordered response sequencing with variable spatial arrays but no effect with a fixed spatial array. Intra-vlPFC infusions of 5HT_2A_ and DA D_2_-R antagonists affected different aspects of sequencing performance, hypothetically relating to planning and error feedback, respectively. The findings are consistent with a higher order executive function of the vlPFC by which a general rule or strategy is applied to optimise performance for tasks with variable requirements.

## Figures and Tables

**Table 1:** Study subjects and task/drug allocation. Table shows the subject number and corresponding symbol, sex and the tasks completed with corresponding drug manipulation and the number of trials per block in the 4-block task (experiment 1).

			Experiment 1 - 4 Block variable array sequencing task				Experiment 2 - Contrasting the effects of variable and fixed sequences	
Subject number/Symbol	Sex	vlPFC Cannula	Trials per block	vlPFC inactivation using GABA_A_ and GABA_B_ receptor agonists	vlPFC 5HT_2A_ receptor blockade	vlPFC D_2_ receptor blockade	vlPFC inactivation on variable array sequencing task	vlPFC inactivation on fixed array sequencing task
1 	M	✓	16	✓	✓	✓		
2 	M	✓	10	✓	✓	✓		
3 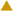	F	✓	12	✓	✓	✓		
4 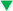	M	✓	14	✓				
5 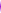	F	✓	10	✓	✓	✓		
6 	F	✓					✓	✓
7 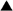	M	✓					✓	✓
8 	F	✓					✓	✓

**Table 2:** Doses used for the M100907 infusion. The column Dose indicates the mass of drug delivered via infusion into the vlPFC calculated by the concentration and volume infused. One dose (1 μg) was given twice but with different concentration and volume. A tick mark indicates that the subject was administered the dose while a blank cell indicates dose was not administered. An x indicates that the dose was too high, the animal disengaged from responding towards the end of the task.

m100907 doses and infusions						
Dose (μg)	Concentration	Volume	Subject 1	Subject 2	Subject 3	Subject 5
0	Vehicle	0.5 μl	**✓**	**✓**	**✓**	**✓**
0.25	0.5 μg/μl	0.5 μl	**✓**		**✓**	
0.5	1 μg/μl	0.5 μl	**×**	**✓**		**✓**
1	2 μg/μl	0.5 μl		**✓**	**✓**	**✓**
0	Vehicle	1 μl		**✓**	**✓**	**✓**
1	1 μg/μl	1 μl		**✓**	**✓**	**✓**
1.5	1.5 μg/μl	1 μl		**✓**		**✓**
2	2 μg/μl	1 μl		**×**	**✓**	**×**

## Significance statement

This investigation employing local intracerebral infusions to inactivate the lateral prefrontal cortex of the New World marmoset reveals the important role of this region in self-ordered response sequencing in variable but not fixed spatial arrays. These novel findings emphasize the higher order functions of this region, contributing to cognitive flexibility and planning of goal directed behavior. The investigation also reports for the first time somewhat contrasting neuromodulatory deficits produced by infusions of dopamine D_2_ and 5-HT_2A_ receptor antagonists into the same region, of possible significance for understanding cognitive deficits produced by anti-psychotic drugs.
